# SU8 etch mask for patterning PDMS and its application to flexible fluidic microactuators

**DOI:** 10.1038/micronano.2016.45

**Published:** 2016-09-12

**Authors:** Benjamin Gorissen, Chris Van Hoof, Dominiek Reynaerts, Michael De Volder

**Affiliations:** 1 Department of Mechanical Engineering, Katholieke Universiteit Leuven & Flanders Make, Celestijnenlaan 300B, 3001 Leuven, Belgium; 2 Imec, Kapeldreef 75, 3001 Leuven, Belgium; 3 Institute for Manufacturing, Department of Engineering, University of Cambridge, 17 Charles Babbage Road, Cambridge, CB3 0FS, UK

**Keywords:** PDMS lithography, SU8, etch mask, microactuator, bending actuator, fluidic actuator

## Abstract

Over the past few decades, polydimethylsiloxane (PDMS) has become the material of choice for a variety of microsystem applications, including microfluidics, imprint lithography, and soft microrobotics. For most of these applications, PDMS is processed by replication molding; however, new applications would greatly benefit from the ability to pattern PDMS films using lithography and etching. Metal hardmasks, in conjunction with reactive ion etching (RIE), have been reported as a method for patterning PDMS; however, this approach suffers from a high surface roughness because of metal redeposition and limited etch thickness due to poor etch selectivity. We found that a combination of LOR and SU8 photoresists enables the patterning of thick PDMS layers by RIE without redeposition problems. We demonstrate the ability to etch 1.5-μm pillars in PDMS with a selectivity of 3.4. Furthermore, we use this process to lithographically process flexible fluidic microactuators without any manual transfer or cutting step. The actuator achieves a bidirectional rotation of 50° at a pressure of 200 kPa. This process provides a unique opportunity to scale down these actuators as well as other PDMS-based devices.

## Introduction

Polydimethylsiloxane (PDMS) is one of the most versatile materials for fabricating microsystems^1^. Simple replication molding^2^ allows replicating features as small as 0.4 nm (Ref. 3) and structures with aspect ratios exceeding 50:1 (Ref. 4). Furthermore, PDMS can be bonded to itself, silicon wafers, and glass slides by a straightforward oxygen–plasma process^5^. These properties have been key for scientific progress in important fields of research, including microfluidics and imprint lithography. Although most current PDMS devices are fabricated using replication molding, emerging domains such as soft robotics^6–9^ require, on one hand, the ability to shape PDMS by molding or by selective curing and, on the other hand, the ability to locally remove PDMS. The latter is currently often performed manually by locally cutting away material with a scalpel. This process is both inaccurate and slow, and therefore, more integrated lithography-based processes using etching would enable further scaling down of soft robotic systems to micrometer sizes to enable new applications of PDMS devices.

Several research groups demonstrated the ability to dry etch PDMS using fluorine-based plasmas that are able to break down the Si–O backbone of PDMS^10,11^. However, the aluminum and gold hardmasks that are used cause high surface roughness, most likely by re-sputtering the etch mask material^12^ and a mismatch in the thermal expansion coefficient between PDMS and the metal mask^13^.

Instead of metal etch masks, we suggest using SU8 photoresist (MicroChem, Westborough, MA, USA) as an etch mask in conjunction with a sacrificial release layer. Both SU8 and PDMS are etched by RIE with a mixture of CF_4_/SF_6_ and O_2_, but the gas composition for efficient PDMS etching is different for SU8 (Ref. 14). Furthermore, an important advantage of SU8 masks is that they can be patterned in thick layers (>200 μm) with aspect ratios over 20 (Ref. 15), allowing long etch times. A disadvantage of SU8 masks is that they are very difficult to remove after the etching process. Thus, we developed a process using a thin sacrificial lift-off resist (LOR; MicroChem) layer that is etched afterwards to release the SU8 masks. Previous research^16^ suggests using SU8 as a mask but provides no solution for the removal of the etch mask. By introducing the sacrificial LOR-layer, the SU8 masking layer can be easily removed, which is important for most applications because the SU8 mask or over-etching are undesirable.

A typical example of a soft robotic device requiring structuring of PDMS is flexible pneumatic bending actuators that are used to execute delicate tasks such as handling biological tissues that is impossible using traditional rigid robots^8,17–21^. These actuators show a large bending deformation when pressurized and is used as a demonstrator in this paper. In their most straightforward configuration, they consist of an inflatable void surrounded by an asymmetric flexible structure consisting, for instance, of two bonded PDMS layers with different thicknesses^22^. To date, these actuators are typically fabricated by a combination of replication molding and manual cutting; this type of fabrication limits the size of the actuators as well as the fabrication throughput. Here we demonstrate how the proposed SU8/LOR etch mask can be used to replace this manual process, thus enabling opportunities for further miniaturization of these PDMS devices.

## Materials and methods

In the literature, a fluorine-based plasma has been suggested to dismantle the silicon-oxygen backbone of PDMS making it possible to etch it with typical etch parameters summarized in Table 1. Vlachopoulou *et al.*^23^ used pure SF_6_ as an etchant and yielded an etch rate of 48 μm h^−1^. The addition of O_2_ to the etch gas was found to decrease the PDMS etch rate. However, Garra *et al.*^12^, Oh *et al.*^11^, Bjørnsen *et al.*^24^, and Szmigiel *et al.*^25^ indicated that a small amount of O_2_ allows an increase in the etch rate. According to Oh, O_2_ might increase the number of reactive fluorine atoms present in the plasma. Szmigiel, however, stated that O_2_ is used to activate the surface of PDMS because of oxidation of the methyl-groups in PDMS:

$$\;C_{x}H_{y}\left(solid\right)+O_{2}\left(gas\right)\overset{\mathrm{plasma}}{\to }CO\left(gas\right)+H_{2}O(gas)$$

These authors also showed that there is a positive correlation between etch rate and reactor power. An overall maximum etch rate (72 μm h^−1^) was achieved by Szmigiel *et al.*^25^ using a 3:1 ratio of SF_6_ to O_2_. Alternatively, Tserepi *et al.*^26^ used SF_6_ together with inert He to achieve an etch rate of 72 μm h^−1^.

The most commonly used hardmask for RIE processing of PDMS is aluminum^12,23,25^. Using this hardmask, poor surface roughness of both the exposed and non-exposed PDMS parts was observed. The exposed PDMS was deteriorated by resputtering the aluminum-masking layer, and excessive wrinkling could be seen on the masked PDMS because of a mismatch in the thermal expansion coefficient, as reported by Cristea *et al.*^13^. In another approach, normal photoresists were used as a masking layer^11,26^. These resist layers were all affected by the RIE process with a selectivity ranging between 4.5 and 0.09 (etch rate PDMS/etch rate masking material). Because masking layer thicknesses are on the order of a few micrometers, only limited layer thicknesses of PDMS can be etched before the photoresist etch masks deteriorated.

**Figure 1:**
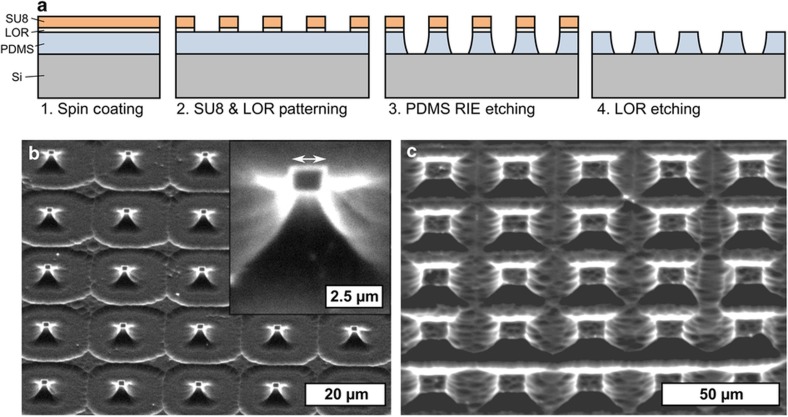
(**a**) Process overview of RIE etching of PDMS using an LOR/SU8 etching mask. (**b**) Tilted SEM pictures (40°) of the smallest features produced by this RIE etching process with a top edge length of 1.5 μm (arrow) showing slanted sidewalls. (**c**) Tilted SEM pictures (40°) of features produced using this RIE etching process, showing the need for sufficient spacing between features because of the slanted sidewalls. RIE, reactive ion etching; SEM, scanning electron microscopy.

To etch thick layers of PDMS while maintaining a good surface roughness, SU8/LOR is proposed in this paper as a masking layer. SU8 has the advantage that it can provide thick high aspect ratio masks. Because SU8 consists of a chain of hydrocarbon bonds, it will be affected by the oxygen plasma^27^. In optimal conditions (5% SF_6_ and 95% O_2_), a SU8 etch rate of 120 μm h^−1^ can be achieved^14^; however, this etch ratio shows a steep decline as the volume percentage of SF_6_ increases, which is then in turn very effective for etching PDMS. This difference in optimal gas composition makes SU8 a good candidate for the masking material for PDMS reactive ion etching using a large excess of SF_6_ over O_2_. However, because SU8 is such a resilient material, we found it difficult to remove after the RIE etch, and therefore, a thin sacrificial LOR layer is applied under the SU8 mask to lift it off after the RIE step as shown in Figure 1a. Obviously, this LOR layer can be omitted if the SU8 layer thickness is entirely consumed at the end of the RIE step. This, however, requires very careful control of the etching time, as well as over the PDMS and the SU8 thickness.

## Results and discussion

### Reactive ion etching of PDMS

To determine the opportunities and limitations of the process above, we first processed a range of pillars with different dimensions and spacing in order to establish the minimal feature size that can be achieved by this process. For this, a thin PDMS layer (Sylgard 184, 10:1) is spin coated at 6000 r.p.m. (5 μm) and is coated by an etch mask consisting of a layer of LOR1A spin coated at 1000 r.p.m. (0.2 μm) and a layer of SU82002 spin coated at 2000 r.p.m. (2.4 μm). The latter is patterned by ultraviolet lithography to define pillars with a square cross section. Etching parameters were 1:4 of O_2_:SF_6_ at a pressure of 150 mtorr, total gas flow rate of 95 sscm and an RIE power of 300 W, for 2×10 min. Figure 1b shows an SEM picture of the smallest features achieved under these test conditions. These pillars have a top edge length of 1.5 μm, increasing in cross section towards the base. These slanted sidewalls have also been reported by Szmigiel *et al.*^25^ and can be made steeper by lowering the etching pressure at the cost of slower etch rates. These sloped edges also limit the minimal spacing of features, as illustrated in Figure 1c, where a spacing of 20 μm was required to create separated PDMS structures. Improvements in the aspect ratio of the structures will be needed for applications requiring closely spaced PDMS features.

Our etching experiments showed a PDMS etch rate of 51 μm h^−1^ and an SU8 etch rate of 15 μm h^−1^, resulting in a process selectivity of 3.4. Specifically, SU8 masks should be about one-third the thickness of the PDMS layer to provide a sufficient etch barrier while retaining good resolution. Finally, our process resulted in clean top surfaces in contrast to previous publications^11,21,23^ and our own experiments using metal hard masks in the same etcher, as shown in Supplementary Figure S1. This figure compares top surfaces using aluminum hard masks (Supplementary Figure S1a) to LOR-SU8 masks on a thick PDMS layer (Supplementary Figure S1b).

### Soft microactuator demonstrator

**Figure 2:**
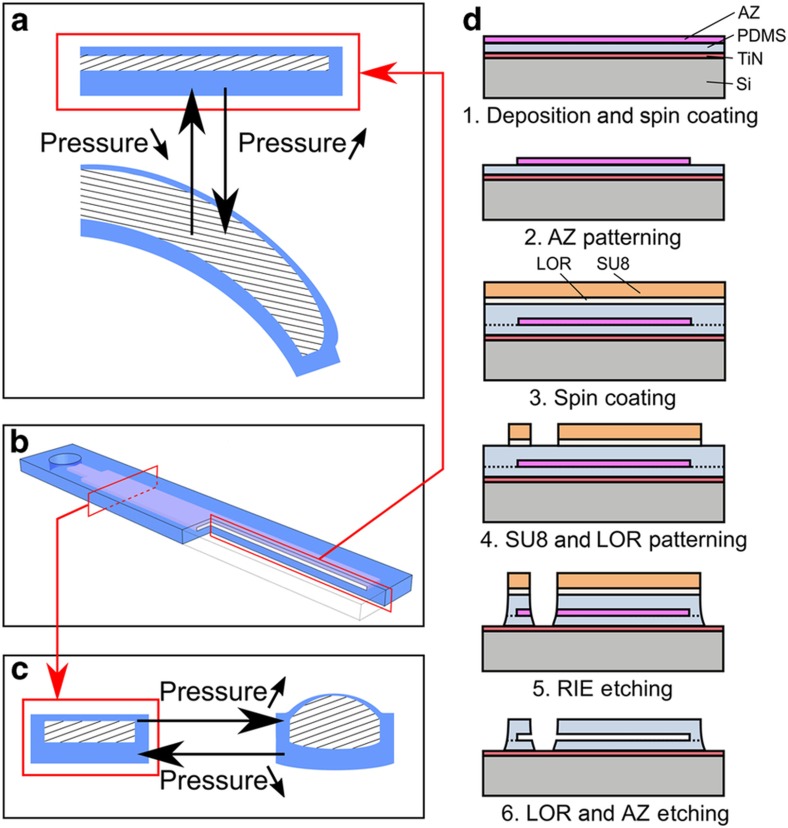
(**a**) Schematic overview of the longitudinal deformation of a flexible bending actuator fabricated using only lithography process steps. This actuator essentially consists of an asymmetric void (hatched) surrounded by a highly flexible material (blue). (**b**) General 3D topology of a soft bending actuator that consists of an internal void between two layers of PDMS that can be inflated through a pressure supply hole. A quarter of the actuator is removed to show cross-sectional cuts. (**c**) Schematic overview of the cross-sectional deformation of a flexible bending actuator, showing its rectangular topology. (**d**) Overview of the full lithographical process to produce these actuators, using RIE etching of PDMS with a LOR/SU8 masking layer to define the outer contours. PDMS, polydimethylsiloxane; 3D, three-dimensional.

To demonstrate the opportunities offered by this etching technology, a lithography production process was developed for fabricating flexible fluidic actuators. These actuators use fluid pressure to inflate closed volumes that cause a highly elastic surrounding structure to deform. Previous research has shown that bending^28^, twisting^29^, and elongation or contraction^30^ can be achieved by these actuators. Because of their compliancy, these actuators can be used to handle delicate objects or can be used in surgical operations^31^. The actuator described in this paper exhibits a large bending deformation when pressurized. This deformation is achieved by inflating a void between two layers of PDMS with different thicknesses as depicted schematically in Figures 2a–c; this principle has been extensively discussed in the literature^28,32^.

Here we focus on a new production flow for these actuators (Figure 2d). First, a 70-nm TiN layer is deposited to prevent PDMS from sticking to the Si wafer and to ease the removal of the actuators at the end of the process. A first layer of PDMS (Sylgard 184, 10:1) is spin coated at 3000 r.p.m., resulting in a layer thickness of ≈37 μm. Then, a sacrificial layer is deposited and patterned to create the internal channels and voids according to the actuator design. The material used for this sacrificial layer is AZ 4562 (MicroChem); then, the material is spin coated at 2000 r.p.m. and patterned to form a rectangular void with a thickness of ≈10 μm. To seal the void, another layer of PDMS was spin coated at a speed of 6000 r.p.m., resulting in an average thickness of ≈23 μm, leading to a local layer thickness on top of the sacrificial structure of ≈13 μm. The thickness ratio of the PDMS layers was chosen in the range of 2 to 3, because this range leads to optimal actuation performance^28^. The combination of the two previous steps makes it possible to define the inner structures of the actuator without having to manually position the two layers relative to each other, as was required in previous research^33^.

The RIE of PDMS defines the outer contours of the actuators where the combination of LOR30B/SU8 2050 is used as an etch mask, as described above. SU8 is spin coated at 2000 r.p.m. to have a layer thickness of ≈57 μm and is patterned afterwards. When taking into account the previously determined selectivity, this layer should be more than thick enough to protect the underlying PDMS. PDMS etching was performed using a 1:4 volume ratio of O_2_ to SF_6_ because Szmigiel *et al.*^25^ considered this ratio to be a near optimal ratio for PDMS fast etching. At a pressure of 150 mtorr and with an RIE power of 300 W, etching was performed for 9×10 min to ensure that PDMS was fully etched away where no masking layer was present. By etching away the sacrificial LOR layer in a developer (OPD5262), the SU8 layer is removed through lift-off. The RIE process also opened the pressure connection hole that is needed for pressurization and wet etching of the sacrificial layer between both PDMS layers to form the inflatable void. This last wet-etching step was performed using acetone that introduced a temporarily light amount of swelling that disappeared after acetone evaporation^34^. SEM pictures that are taken during this production process are shown in Supplementary Figure S2.

**Figure 3:**
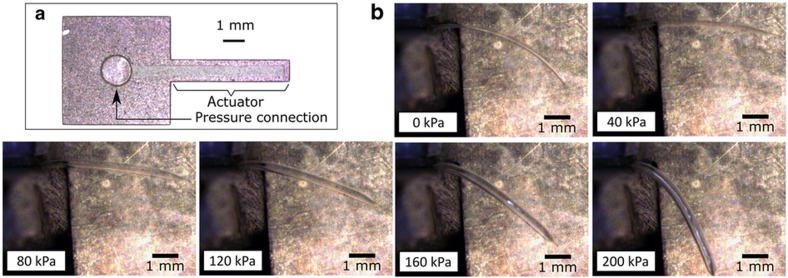
(**a**) Top view of a flexible fluidic actuator with a highlighted inflatable void that was fabricated using a purely lithographical production process. The outer dimensions of the actuator are 5.5 mm×1 mm, with PDMS layers of ≈37 and ≈13 μm and an inflatable void height of ≈10 μm. (**b**) Bending deformation of this flexible fluidic actuator upon pressurization up to a pressure of 200 kPa. PDMS, polydimethylsiloxane.

The top view of a flexible fluidic actuator (with planar dimensions of 5.5 mm×1 mm and an inflatable void of 5.25 mm×0.5 mm) that was made using this production process can be seen in Figure 3a. Figure 3b shows the deformation of this actuator when pressurized to 200 kPa. As illustrated in these consecutive pictures, the actuator shows a typical bidirectional bending motion; that is, at low pressures we first observe a small bending deformation at the side of the thin PDMS layer and at larger pressures, a bending deformation towards the other side that is typical for this type of actuator^28,33,35^. Overall, a bending stroke of 50° is observed between 40 and 200 kPa. It is worth noting that in our experience, 200 kPa is higher than the pressures achieved for micromolded actuators that typically failed at lower pressures (80–150 kPa)^28^. This could be attributed to the fact that the bonding between PDMS layers is better for the lithographical case (liquid PDMS on solid PDMS) than in the micromolded case (solid PDMS bonding on solid PDMS using oxygen plasma activation). The potential to operate at a high pressure is an important advantage of the developed process for pneumatic microrobots and also includes the potential for high-pressure microfluidics.

## Conclusion

PDMS has become omnipresent in microsystems technology and has been particularly instrumental for the development of microfluidic systems. Although PDMS is easy to pattern by molding, it is very difficult to etch. Advances have been made in etching PDMS with metal hardmasks, but the resilience of PDMS against RIE (O_2_:SF_6_) results in the need for very thick metal masks. In addition, this process suffers from metal resputtering during the etching process. Polymer masks have been suggested in the literature; however, to process thicker PDMS layers with higher quality, we suggest using SU8 hardmasks. SU8 is a well-established photoresist that can easily be patterned to obtain high aspect ratio masks that withstand the RIE process. This affords the opportunity to etch thick PDMS layers; however, SU8 has the disadvantage of being difficult to strip away after the RIE patterning step. We solve this issue by using a sacrificial LOR layer to remove the SU8 mask.

We further demonstrate how this process can be used for fabricating smaller flexible fluidic microactuators. Previous production processes to make these actuators involved a manual production step that made accurate positioning impossible. In this paper, a new production process is presented that only uses lithographical techniques; this process makes it possible for dimensions to shrink down to the micrometer range. As a demonstrator, a flexible fluidic actuator was fabricated that exhibits a bidirectional bending motion of 50° and is able to withstand pressures of up to 200 kPa.

## Figures and Tables

**Table 1:** Literature overview of RIE of PDMS

RIE gasses	Gas ratio	Etch rate (μm h^−1^)	Mask
SF_6_	—, Vlachopoulou^23^	48	Aluminum
CF_4_:O_2_	3:1, Garra^12^	20	Aluminum
	1:1, Oh^11^	60	AZ9260
SF_6_:O_2_	4:1, Bjørnsen^24^	30	Glass slide
	3:1, Szmigiel^25^	72	Aluminum
He:SF_6_	95:5, Tserepi^26^	72	AZ5214

Abbreviations: PDMS, polydimethylsiloxane; RIE, reactive ion etching.
