# Supplementary material for: SU8 etch mask for patterning PDMS and its application to flexible fluidic microactuators
Source: Microsyst Nanoeng. 2016 Sep 12;2:16045. doi: 10.1038/micronano.2016.45 (PMC6444735; doi:10.1038/micronano.2016.45)
Supplement: Supplementary Information [file micronano201645-s1.pdf]

Supplementary file

SU8 etch mask for patterning PDMS and its application to flexible fluidic microactuators

Benjamin Gorissen[1], Chris Van Hoof[2], Dominiek Reynaerts[1] and Michael De Volder[1,3]

Microsystems & Nanoengineering (2016) **2,** 16045; doi:10.1038/micronano.2016.45; Published online: 12 September 2016

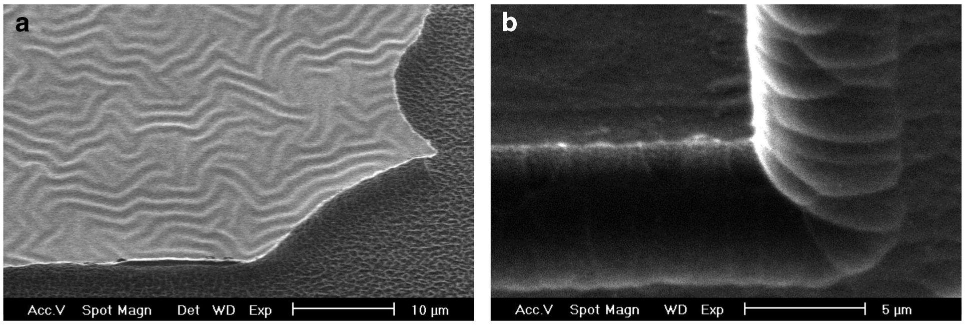

Figure S1 (**a**) Tilted SEM picture of a PDMS structure RIE etched using an aluminum hardmask, showing wrinkling of the top surface due to a mismatch in thermal expansion coefficient. (**b**) Tilted SEM picture of a PDMS structure RIE etched using the LOR/SU8 mask, showing a clean top surface.

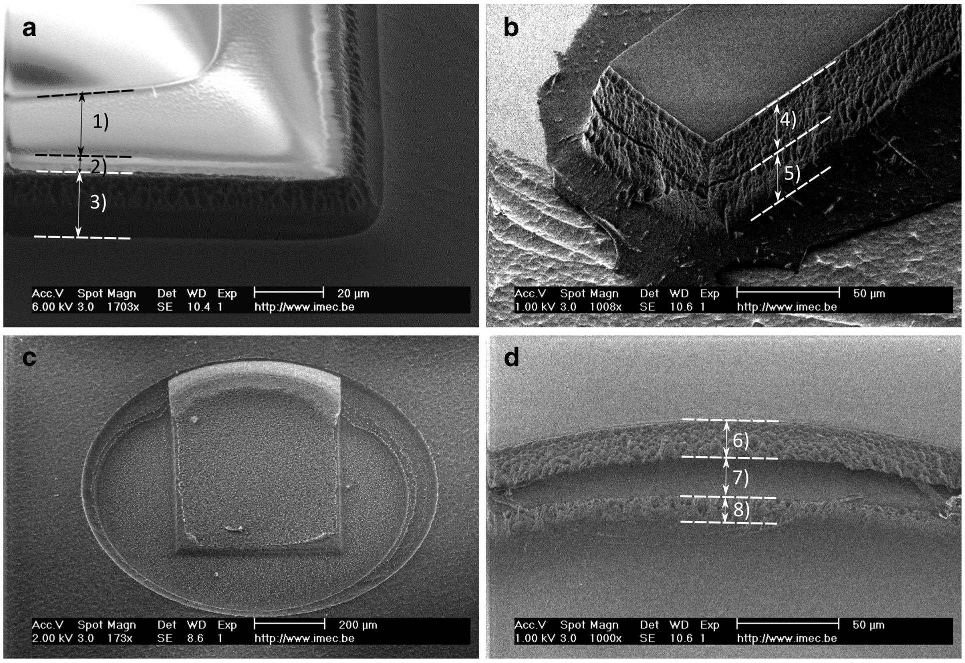

Figure S2 Tilted SEM pictures of the production process. (**a**) Side view of an O_2:SF_6 etched PDMS structure, showing the SU8 masking layer (1), LOR release layer (2) and PDMS vertical side walls (3). (**b**) Image of the tip of a PDMS actuator after O_2:SF_6 etching and wet etching of the LOR layer, clearly showing the thin PDMS layer (4) and thick PDMS layer (5) the actuator is composed of. (**c**) Image of the pressure supply hole, clearly showing the sacrificial layer between the two layers of PDMS being partially etched away by the RIE process. (**d**) Detailed view of the pressure supply hole after wet etching of the sacrificial layer between the two layers of PDMS (6) and (8), showing the void were the sacrificial layer used to be (7).

[1]Department of Mechanical Engineering, Katholieke Universiteit Leuven & Flanders Make, Celestijnenlaan 300B, 3001 Leuven, Belgium; [2]Imec, Kapeldreef 75, 3001 Leuven Road, Cambridge, MA CB3 0FS, USA and [3]Institute for Manufacturing, Deptartment of Engineering, University of Cambridge, 17 Charles Babbage Road, Cambridge, CB3 0FS, UK.
Correspondence: Michael De Volder (michael.devolder@kuleuven.be or mfld2@cam.ac.uk)
