# Supplementary figures and images for: SU8 etch mask for patterning PDMS and its application to flexible fluidic microactuators
Source: Microsyst Nanoeng. 2016 Sep 12;2:16045. doi: 10.1038/micronano.2016.45 (PMC6444735; doi:10.1038/micronano.2016.45)

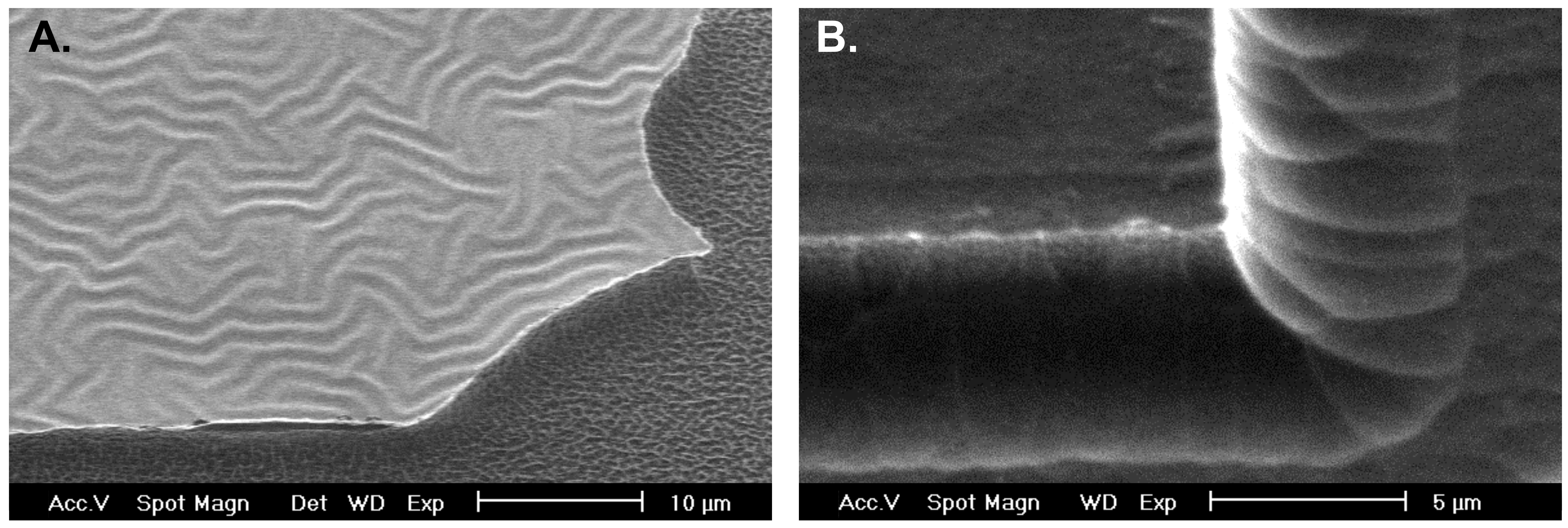

Supplement: Supplementary Figure S1 [file micronano201645-s2.tif]

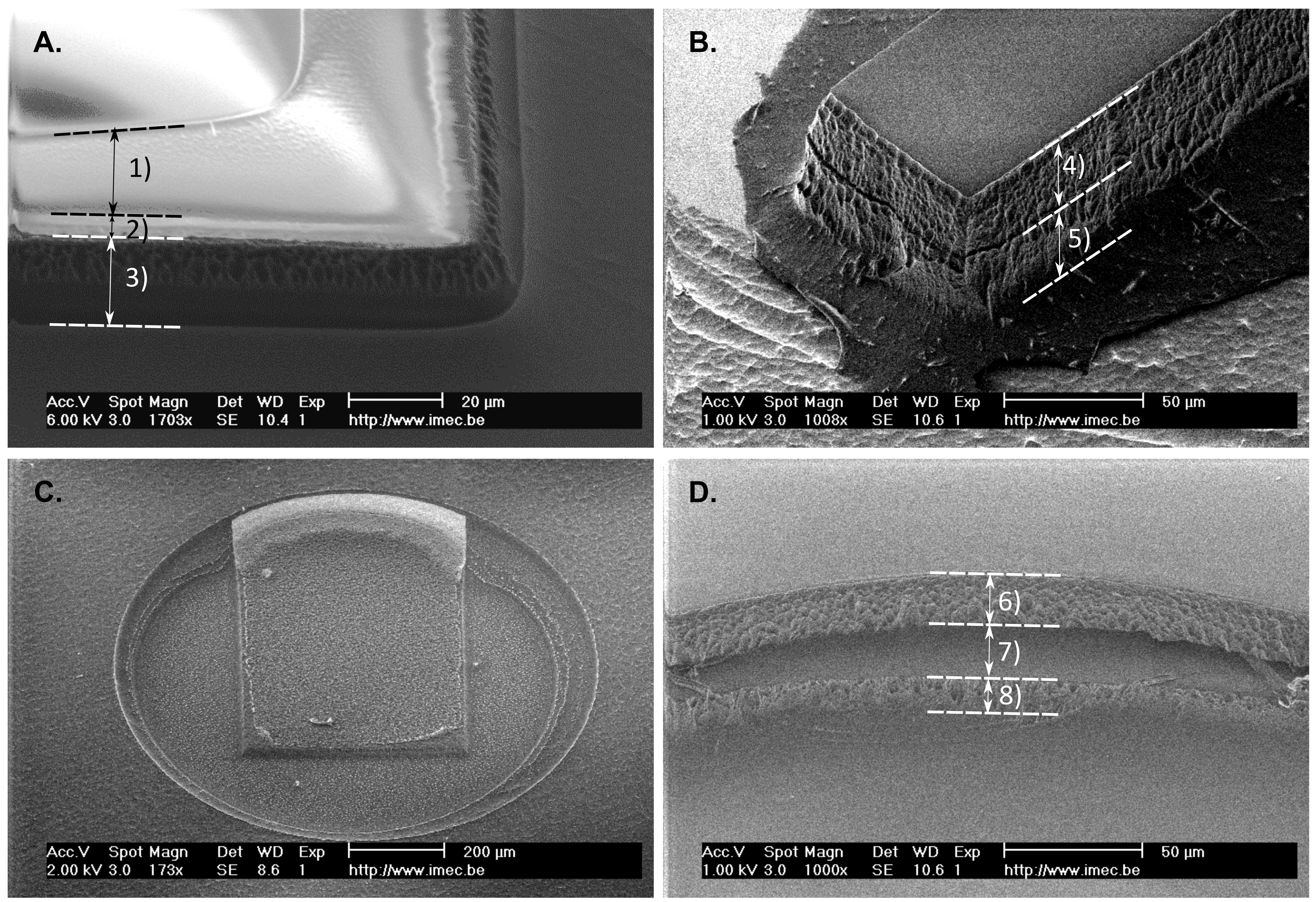

Supplement: Supplementary Figure S2 [file micronano201645-s3.tif]
